# High-risk *Pseudomonas aeruginosa* clones harboring β-lactamases: 2024 update

**DOI:** 10.1016/j.heliyon.2024.e41540

**Published:** 2024-12-26

**Authors:** Verónica Roxana Flores-Vega, Santiago Partida-Sanchez, Miguel A. Ares, Vianney Ortiz-Navarrete, Roberto Rosales-Reyes

**Affiliations:** a Unidad de Medicina Experimental, Facultad de Medicina, Universidad Nacional Autónoma de México, Mexico City, Mexico; b Departamento de Biomedicina Molecular, Centro de Investigación y de Estudios Avanzados del IPN, Mexico City, Mexico; c Center for Microbial Pathogenesis, Abigail Wexner Research Institute at Nationwide Children's Hospital, Columbus, OH, USA; d Department of Pediatrics, College of Medicine, The Ohio State University, Columbus, OH, USA; e Unidad de Investigación Médica en Enfermedades Infecciosas y Parasitarias, Hospital de Pediatría, Centro Médico Nacional Siglo XXI, Instituto Mexicano del Seguro Social, Mexico City, Mexico

**Keywords:** *Pseudomonas aeruginosa*, Sequence type, High-risk, Multidrug resistance, β-Lactamase genes

## Abstract

Carbapenem-resistant *Pseudomonas aeruginosa* is defined by the World Health Organization as a “high priority” in developing new antimicrobials. Indeed, the emergence and spread of multidrug-resistant (MDR) or extensively drug-resistant (XDR) bacteria increase the morbidity and mortality risk of infected patients. Genomic variants of *P. aeruginosa* that display phenotypes of MDR/XDR have been defined as high-risk global clones. In this mini-review, we describe some international high-risk clones that carry β-lactamase genes that can produce chronic colonization and increase infected patients' morbidity and mortality rates.

## 1 Introduction

*P. aeruginosa* is a non-fermenting Gram-negative rod. This bacterium can colonize diverse environments, including aquatic and soil habitats [1]. This opportunistic pathogen also can produce acute and chronic infections in humans and animals [2]. In humans, *P. aeruginosa* causes nosocomial infections, particularly in patients affected by immunocompromise, with life-threatening illnesses and injuries in the Intensive Care Unit (ICU) [3], or individuals affected by some chronic lung disease, such as Cystic Fibrosis [4].

*P. aeruginosa* can carry genes coding for antibiotic resistance; this might result in the emergence of defined high-risk clones [2]. Nevertheless, the genome plasticity of *P. aeruginosa* makes this pathogen metabolically versatile, displaying an ability to adapt to different environmental conditions [5]. Genetic diversity arises from new mutations when the bacterium is exposed to aggressive environments [[6], [7], [8]]. The cumulative mutations in their genome could result in bacterial micro-evolution [9,10]. Strains under these conditions increase the chance of emerging as new bacterial variants, changing the expression of some virulence factors and raising antibiotic resistance profile [[11], [12], [13], [14]]. These bacterial variants can also display new metabolic and cellular morphologies with better survival chances in hostile environments [[6], [7], [8]]. Efficiently identifying these bacterial variants and their follow-up could help physicians establish better strategies to avoid their widespread among susceptible individuals.

## 2 Classification of genomic variants of *P. aeruginosa*

The World Health Organization (WHO) has defined *P. aeruginosa* with carbapenem resistance as a member of the high-priority group for developing new antimicrobials [15]. Some variants of *P. aeruginosa* have been defined as high-risk clones, particularly those causing nosocomial infections, and their spread among hospitalized individuals. Molecular epidemiology surveillance helps to identify the emergence, incidence, and prevalence of high-risk clones; this information helps to decrease their spread worldwide. Some strategies to identify high-risk clones have been developed, and one of the most valuable strategies is the Multi-Locus Sequence Typing (MLST). MLST is a sequence-based genotyping method used to determine specific genomic bacterial variants; MLST requires the analysis of sequences ∼400 pb long of seven or more specific gene *loci*. These regions are amplified by PCR and sequenced to determine the allele and bacteria type based on a set of housekeeping alleles available on the PubMLST website (https://pubmlst.org/organisms/pseudomonas-aeruginosa/) [16]. Thus, MLST has been established as a standard method worldwide to perform molecular epidemiology surveillance of *P. aeruginosa* [[17], [18], [19]].

## 3 Identification of high-risk sequence types of *P. aeruginosa*

The increase in the frequency of nosocomial infections caused by *P. aeruginosa* multidrug-resistant (MDR), defined as acquired non-susceptibility to at least one agent in three or more antimicrobial categories, extensively drug-resistant (XDR); defined as non-susceptibility to at least one agent in all but two or fewer antimicrobial categories, and sometimes by pan-drug resistant (PDR) defined as non-susceptibility to all agents in all antimicrobial categories [20] compromise the selection of effective treatments with available antimicrobials. *P. aeruginosa* develops resistance to almost all available antimicrobials and adapts to hostile environments by selecting chromosomal mutations [21]. In addition, antimicrobial resistance can also increase by incorporating exogenous genes carried in mobile elements (integrons encoded in transferable genetic elements as plasmids or transposons) [22]. Lactams are one of the most valuable families of antimicrobials used in patients colonized with *P. aeruginosa*. Nevertheless, several clinical isolates carry genes that code β-lactamases with a greater hydrolytic spectrum [extended-spectrum-β-lactamases (ESBLs) and carbapenemases] [23,24]. The emergence of high-risk clones with MDR or XDR phenotypes represents a serious global health threat [25]. Colistin (CST), a last-resort antibiotic for treating MDR or XDR pathogens [[26], [27], [28]], has seen increasing resistance rates. Meta-analysis reveals that global CST resistance increased from 2 % (2006–2010) to 5 % (2020–2023) [29]. Among patients with Cystic Fibrosis, frequently colonized by *P. aeruginosa* exhibiting MDR or XDR phenotypes, CST resistance is particularly concerning, as it significantly increases the frequency of exacerbations and mortality risk [30]. Globally, *P. aeruginosa* MDR/XDR strains belonging to sequence types ST111, ST175, ST233, ST235, and ST244 are recognized as high-risk clones (Table 1) [18,31,32]. In contrast, ST274, ST395, and ST463 are considered locally significant high-risk clones in Spain, France, and China, respectively [31,[33], [34], [35]]. CST resistance has been reported in some high-risk clones, including ST111, ST175, ST233, ST235, ST244, and ST463.

**Table 1:** β-Lactamase genes carried by high-risk sequence types of *P. aeruginosa.*

Clone			*Enzymes*
ST111	*β-lactamase*	Class A	*bla*_CTX-M-like_, *bla*_GES-7_, *bla*_KPC-2_, *bla*_CARB-2(PSE-1)_ *bla*_CARB-1(PSE-4)_, *bla*_CARB-3_, *bla*_TEM-like_, and *bla*_VEB-1_.
		Class B	*bla*_GIM-1_, *bla*_IMP-1_, *bla*_IMP-4_, *bla*_IMP-7_, *bla*_IMP-9_, *bla*_IMP-13_, *bla*_IMP-18_, *bla*_IMP-29_, *bla*_IMP-33_, *bla*_IMP-65_, *bla*_NDM-1_, *bla*_VIM-1_, *bla*_VIM-2_, and *bla*_VIM-11_.
		Class D	*bla*_OXA-2_, *bla*_OXA-4_, *bla*_OXA-9_, *bla*_OXA-10_, *bla*_OXA17_, *bla*_OXA-46_, *bla*_OXA-74_, *bla*_OXA-101_, *bla*_OXA-142_, *bla*_OXA-181_, *bla*_OXA395_, and *bla*_OXA-520_.
	T3SS	Exotoxins	*exoS, exoT,* and *exoY*.
		Countries	Australia, Belgium, Bulgaria, Brazil, China, Canada, Colombia, Costa Rica, Croatia, Denmark, France, Germany, Greece, Holland, Hungary, Iran, India, Italy, Korea, Lebanon, Mexico, Netherland, Norway, Panama, Peru, Poland, Portugal, Russia, Saudi Arabia, South Africa, Spain, Sweden, Sudan, Thailand, Tunisia, Turkey, UK, USA, and Venezuela.
		References	[32,36,50,[94], [95], [96], [97], [98], [99], [100], [101], [102], [103], [104], [105], [106], [107], [108], [109], [110], [111], [112], [113], [114], [115], [116], [117], [118], [119], [120], [121], [122], [123]].
ST175	*β-lactamases*	Class A	*bla*_AER-1_, *bla*_CARB-12_, *bla*_GES-1_, *bla*_GES-5_, and *bla*_TEM-1A_.
		Class B	*bla*_IMP-1_, *bla*_IMP-7_, *bla*_IMP-8_, *bla*_IMP-13_, *bla*_IMP-18_, *bla*_IMP-19_, *bla*_VIM-1_, *bla*_VIM-2_, *bla*_VIM-8_, and *bla*_VIM-20_.
		Class D	*bla*_OXA-2_, *bla*_OXA-35_, *bla*_OXA-50_, *bla*_OXA-101_, *bla*_OXA-210_, and *bla*_OXA-681_.
	T3SS	Exotoxins	*exoS, exoT,* and/or *exoY*.
		Countries	Canada, Czech Republic, China, France, Germany, Hungary, Iran, Italy, Japan, Poland, Portugal, Spain, UK, and USA.
		References	[32,34,41,42,45,50,65,94,96,108,110,115,116,119,[124], [125], [126], [127], [128], [129], [130], [131]].
ST233	*β-lactamases*	Class A	*bla*_GES-15_
		Class B	*bla*_IMP-1,_*bla*_IMP-7,_*bla*_IMP-15,_*bla*_NDM-1,_*bla*_VIM-2,_*bla*_VIM-17,_ and *bla*_VIM-48_.
		Class D	*bla*_OXA-4_, *bla*_OXA-50_, *bla*_OXA-396_, and *bla*_OXA-486_.
	T3SS	Exotoxins	*exoS, exoT,* and *exoY*.
		Countries	Australia, Bahrein, Bolivia, Brazil, China, Czech Republic, Denmark, Egypt, France, Germany, Ghana, India, Japan, Ivory Coast, Kenia, Lebanon, Mexico, Nigeria, Norway, Poland, Qatar, Romania, Singapore, Spain, South Africa, Saudi Arabia, UK, and USA.
		References	[32,46,48,50,96,107,111,120,[132], [133], [134], [135], [136], [137], [138], [139], [140], [141], [142]].
ST235	*β-lactamases*	Class A	*bla*_BEL-1_, *bla*_CARB-1(PSE-4)_, *bla*_CARB-2__(PSE-1)_, *bla*_CTXM-2_, *bla*_CTXM-like_, *bla*_GES-1_, *bla*_GES-5_, *bla*_GES-6_, *bla*_GES-7_, *bla*_GES-8__(IBC-2)_, *bla*_GES-9_, *bla*_GES-9-like_, *bla*_GES-19_, *bla*_GES-20_, *bla*_GES-24_, *bla*_KPC-2_, *bla*_PER-1_, *bla*_PER-3_, *bla*_SHV-2_, *bla*_SHV-2a_, *bla*_SHV-5_, *bla*_SMP-1_, *bla*_TEM-1A_, *bla*_TEM-1B_, *bla*_TEM-116_, *bla*_VEB-1,__blaVEB-1a,__blaVEB-2,_*bla*_VEB-5,__blaBVeb-9,_ and *bla*_VEB-like_.
		Class B	*bla*_FIM-1_, *bla*_IMP-1_, *bla*_IMP-4_, *bla*_IMP-6_, *bla*_IMP-7_, blaIM-8, *bla*_IMP-10_, *bla*_IMP-13_, *bla*_IMP-14a_, and *bla*_IMP-15_, *bla*_IMP-19_, *bla*_IMP-26_, *bla*_IMP-29_, *bla*_IMP-31_, *bla*_IMP-34_, *bla*_IMP-43_, *bla*_IMP-51_, *bla*_NDM-1_, *bla*_VIM-1_, *bla*_VIM-2_, *bla*_VIM-4_, *bla*_VIM-6_, *bla*_VIM-11_, *bla*_VIM-13_, *bla*_VIM-17_, *bla*_VIM-28_, *bla*_VIM-37_, *bla*_VIM-47_, *bla*_VIM-48_, *bla*_VIM-54_, *bla*_VIM-62_, and *bla*_VIM-like_.
		Class D	*bla*_LCR-1_, *bla*_NPS-like_, *bla*_OXA-1_, *bla*_OXA-2_, *bla*_OXA-4_, *bla*_OXA-9_, *bla*_OXA-10_, *bla*_OXA-11_, *bla*_OXA-14_, *bla*_OXA-17_, *bla*_OXA-19_, *bla*_OXA-28_, *bla*_OXA-35_, *bla*_OXA-48_, *bla*_OXA-50g_, *bla*_OXA-74_, *bla*_OXA-129_, *bla*_OXA-141_, *bla*_OXA-142_, *bla*_OXA-205_, and *bla*_OXA-488_.
	T3SS	Exotoxins	*exoU, exoT,* and *exoY*.
		Countries	Argentina, Australia, Belarus, Belgium, Brazil, China, Colombia, Croatia, Cyprus, Czech Republic, Estonia, France, Germany, Greece, Hungary, India, Iraq, Iran, Indonesia, Italy, Japan, Kazakhstan, South Korea, Libya, Lithuania, Malaysia, Mexico, Nepal, Netherlands, Nigeria, Norway, Philippines, Poland, Portugal, Qatar, Romania, Russia, Saudi Arabia, Serbia, Singapore, Sri Lanka, Spain, Switzerland, Thailand, Tunisia, Turkey, UAE, UK, USA, and Vietnam.
		References	[32,^34^,^47^,^50^,^51^,^61^,^64^,^92^,^96^,[99], [100], [101], [102],^104^,^108^,^110^,^113^,[115], [116], [117],[119], [120], [121], [122], [123],^125^,^130^,^131^,[133], [134], [135],^141^,[143], [144], [145], [146], [147], [148], [149], [150], [151], [152], [153], [154], [155], [156], [157], [158], [159], [160], [161], [162], [163], [164], [165], [166], [167], [168], [169], [170].
ST244	*β-lactamases*	Class A	*bla*_AER-1_, *bla*_CTXM-2_, *bla*_KPC-2_, *bla*_GES-24_, *bla*_PER-1_, *bla*_SHV_-_like_, *bla*_TEM_-_like_, *bla*_TEM-1A_, *bla*_VEB-1_, *bla*_VEB-2_.
		Class B	*bla*_IMP-1_, *bla*_IMP-6_, *bla*_IMP-9_, *bla*_IMP-10_, *bla*_IMP-45_, *bla*_VIM-2_, *bla*_VIM-4_, *bla*_VIM-like_, and *bla*_NDM-1_.
		Class D	*bla*_OXA-1_, *bla*_OXA-2_, *bla*_OXA-10_. *bla*_OXA-35_, *bla*_OXA-50f_, and *bla*_OXA-486_.
	T3SS	Exotoxins	*exoS,* and *exoT.*
		Countries	Algeria, Argentina, Bangladesh, Brazil, Bulgaria, China, Colombia, Czech Republic, Denmark, Estonia, France, Germany, India, Iraq, Ivory Coast, Japan, Kenia, South Korea, Libya, Nepal, Netherlands, Philippines, Poland, Portugal, Russia, Tanzania, Spain, USA, and Vietnam.
		References	[43,50,51,[65], [66], [67],^92^,^96^,^100^,^117^,^125^,[130], [131], [132], [133],^139^,^143^,[148], [149], [150], [151],[171], [172], [173], [174], [175], [176], [177], [178], [179], [180], [181]].
ST274	*β-lactamases*	Class A	*bla*_*AER-1*_, *bla*_*GES*_
		Class B	*bla*_*IMP*_
		Class D	*bla*_*OXA-486*_.
	T3SS	Exotoxins	*exoS*, and *exoT.*
		Countries	Australia, Brazil, China, Ethiopia, France, Japan, Qatar, Netherland, Spain, Russia, USA.
		References	[68,[70], [71], [72], [73],^140^,[182], [183], [184]].
ST395	*β-lactamases*	Class A	Not reported.
		Class B	*bla*_VIM-2_*, bla*_VIM-4_.
		Class D	*bla*_OXA-15_, and *bla*_OXA-488_.
	T3SS	Exotoxins	*exoS* and *exoT*.
		Countries	France, Germany, Greece, Hungary, Poland, Portugal, Spain, Sweden, Tunisia, and USA.
		References	[41,[74], [75], [76], [77], [78],^116^,^127^,^179^,[185], [186], [187], [188], [189]].
ST463	*β-lactamases*	Class A	*bla*_KPC-2_, *bla*_KPC-33_, and *bla*_KPC-90_.
		Class B	*bla*_IMP-6_*, bla*_IMP-45_, and *bla*_AFM-1_.
		Class D	*bla*_OXA-50_, *bla*_OXA-50-like_, *bla*_OXA-246_, and *bla*_OXA-486_.
	T3SS	Exotoxins	*exoS*, *exoT, exoY,* and *exoU***.**
		Countries	China, and Korea.
		References	[79,[81], [82], [83], [84],^86^,[190], [191], [192]]

***ST***, sequence type, ***T3SS***; Type 3 secretion system; ***UAE***, United Arab Emirates; ***UK***, United Kingdom; ***USA***, United States of America.

## 4 The most frequent high-risk clones of *P. aeruginosa*

### 4.1 ST111

This clone was initially named *P. aeruginosa* AG1 (PaeAG1), which belongs to the serotype O12. This clone was isolated from a sputum sample obtained from a patient with pneumonia in the San Juan de Dios Hospital Intensive Care Unit in San José, Costa Rica [36]. ST111 is considered a high-risk clone by its XDR phenotype; it displays resistance to β-lactams, aminoglycosides, and fluoroquinolones. Resistance to CST was reported in Spain [37] and Russia [38] and to polymyxin B (PXB) in Colombia [39]. ST111 carries the metallo-β-lactamase *bla*_VIM-2_ gene; nevertheless, in several studies, it has been reported that this clone harboring several variants of β-lactamases of class A (CTMX-like, GES, KPC, PSE, TEM-like, and VEB), class B (GIM, IMP, and VIM) and class D (OXA) (Table 1). Most clinical isolates identified to date harbor genes for *bla*_*VIM*-2_ and *bla*_*IMP*-18_ into a class 1 integron located in the 57 PaeAG1 island [40]. This clone is highly transmissible among hospitalized individuals and those affected with CF. Their transmissibility can be due to the expression of several virulence factors that promote its adherence (flagella, type IV pili), anti-phagocytosis ability (alginate production), iron uptake (pyochelin and pyoverdine), production of enzymes (phospholipases), biosurfactants (rhamnolipid biosynthesis), and proteases. Importantly, isolates from this clone also carry the *toxA* gene of the T2SS and the T3SS effectors: *exoS*, *exoT*, and *exoY* but not *exoU* [36]. This clone has been identified in several countries in America, Africa, Asia, Europe, and Oceania.

### 4.2 ST175

The clone 05-314 belongs to the serotype O4. This clone was isolated from a wound swab from a hospitalized patient from a urology ward in Hungary [41]. Most ST175 isolates display an MDR phenotype that carries the metallo-β-lactamase *bla*_*VIM*-2_ (Table 1); this gene is in a class 1 integron that also harbors the aminoglycoside modified enzyme (*aac*6′-1b) [42]. ST175 also carries a class 1 integron harboring the *aadB* gene that confers gentamicin and tobramycin resistance [43]. Resistance to CST was reported only in Spain [44]. ST175 remains susceptible to amikacin, piperacillin/tazobactam, and aztreonam [42]. Notably, some ST175 clones show a phenotype of XDR associated with the hyperproduction of AmpC, with inactivation of the porin OprD, high resistance to fluoroquinolones, and overexpression of the efflux pump MexXY [43]. Overall, this clone harbors genes for β-lactamases class A (AER, CARB, GES, and TEM), class B (IMP and VIM), and class D (OXA) (Table 1). ST175 shows a lower level of virulence in the *Caenorhabditis elegans* model in comparison with the ST111 and ST235, probably due to this clone carrying the *exoS*, *exoT*, and/or *exoY* genes of the T3SS but not *exoU* [45]. This clone has been identified in America, Asia, and Europe.

### 4.3 ST233

The clone K34-7 [46] belongs to the serotype O6 [47]. ST233 was isolated from a tracheal secretion in the Intensive Care Unit (ICU) at the Ullevål University Hospital, Norway [46]. ST233 is a high-risk clone that produces carbapenemases that display an XDR phenotype [48]. Resistance to CST was reported in Mexico [48], and intermediate resistance in the USA [49]. This clone seems to be imported from Ghana and is characterized by carrying the *bla*_VIM-2_ gene [46]. Additionally, ST233 harbors genes for β-lactamases class A (GES), class B (IMP, NDM, and VIM), and class D (OXA) (Table 1). ST233 carries the *exoS*, *exoT*, or *exoY* genes [50]. ST233 has been identified in Africa, America, Asia, Europe, and Oceania.

### 4.4 ST235

This clone belongs to the serotype O11 (this serotype is also associated with other high-risk clones, such as ST357, ST308, and ST298); this clone is the most frequently isolated and spread worldwide. ST235 was first identified in 1992 from a burn patient at Hacettepe University Hospital in Ankara, Turkey. It was formally assigned as ST235 in 2007 [51]. ST235 displays an XDR phenotype; this clone carries over 60 different β-lactamase variants of class A (BEL, CTXM, GES, KPC, PER, PSE, SHV, SMP, TEM, and VEB), class B (FIM, IMP, NDM, and VIM) and class D (LCR, NPS, and OXA) (Table 1). A new sub-linage of ST235 harboring the class A *bla*_*SHV2a*_ has been described [52]. Resistance to CST was reported in India [53], Iran [54], Serbia [55], South Korea [56,57], Turkey [58], and intermediate resistance in Australia [59]. Most ST235 isolates did not harbor a CRISPR/CAS system, suggesting that these clones are more susceptible to acquiring exogenous DNA [14]. ST235 carries the T3SS effectors: *exoU*, *exoT*, and *exoY* [60,61]. The expression of ExoU has been well-established to determine a worse outcome in respiratory and bloodstream infections [45,62]. Additionally, this clone also carries several genes to produce pyoverdine, exotoxin A, hemolytic phospholipase C, non-hemolytic phospholipase C, phenazine synthesis, proteolysis and elastolysis, alkaline protease, and alginate [63,64]. This clone has been identified in Africa, America, Asia, Europe, and Oceania.

### 4.5 ST244

This clone belongs to the serotypes O12 and O2. ST244 was isolated from different sources from a hospital in Warsaw, Poland [51]. This clone shares its origin with ST235. Nevertheless, ST244 does not express the *bla*_OXA-74_. ST244 displays an MDR phenotype; this clone carries different β-lactamase variants of class A (AER, CTMX, KPC, SHV-like, TEM, and VEB), class B (IMP, VIM, and NDM), and class D (OXA) (Table 1) [65]. Resistance to CST was reported in Brazil [66] and South Korea [67]. ST244 carries the *exoS* and *exoT* genes but not *exoU*. This clone was identified in America, Africa, Asia, and Europe.

### 4.6 ST274

This clone belongs to serotype O:3. This clone was isolated from a hospital in Spain [68] and is frequently associated with CF patients [69,70]. ST274 is an MDR clone that frequently displays a phenotype of XDR. This clone carries different β-lactamase variants of class A (AER and GES), class B (IMP), class D (OXA) (Table 1), and class C *bla*_*PDC-24*_*, bla*_*PAO*_, *bla*_*PDC-69*_. Additionally, this strain carries *florR_1*, acc(6′)-1b, acc(6′)-II, aac(6′)-31, aacA8/6′)-Ia, aac(6′)-Ib3, acc(6′)-Iae, *aph(3′)-IIb*, *aph(3″)-Ib*, *aph(6)-Id*, *sul2*, *gyrA*, *gyrB*, *parD*, *parC*, *crpP*, *catB7* and *fosA* genes for resistance to other antimicrobials [[71], [72], [73]]*.* ST274 carries genes that code for the toxins *toxA*, *exoS*, and *exoT* and genes for pyoverdine, pyochelin, and T4P synthesis. This clone is endemic to Spain and is identified in Africa, America, Asia, Europe, and Oceania.

### 4.7 ST395

This clone belongs to the serotype O6. It was isolated in 1997 from the nose of a colonized patient in the surgical intensive care unit (SICU) of the University Hospital, Besançon, France [74]. ST395 is an MDR clone that carries several β-lactamase variants of class B (VIM) and class D (OXA) (Table 1). The *bla*_VIM-2_ was detected into a class 1 integron (*intI1-bla*_OXA-10_*-aadB-bla*_VIM-2_*-aadB-bla*_OXA-10_) [75]. Additionally, this clone carries the *aadA6*, *aph*(3′)*-IIb*, and *aph*(3′)*-Via*, *catB7,* and the Type I-E CRISPR/Cas System [76]. ST395 carries genes that code for the toxins *toxA*, *exoS*, *exoT*, and *exoY*: notably, this clone has a 131 kb chromosomal deletion, in which genes for bacterial adherence, biofilm formation, and genes that participate in the synthesis of type IV pili are missing [77], these findings suggest a lower virulence profile in comparison with a reference strain. Nevertheless, this clone carries genes for biosynthesis of the coenzyme B12, flagella, and quorum sensing [74]. Significantly, ST395 induces overexpression of PD-L1 on monocytes, decreasing the immune response mediated by T-cells [78]. This clone became a dominant endemic clone in France and might be important to colonize and persist in patients affected with CF. ST395 was spread to countries in America, Asia, and Europe.

### 4.8 ST463

This clone belongs to the O4 serotype, identified in 2015 in Hangzhou**,** China [79]. ST463 is an XDR clone with higher resistance to β-lactams, including carbapenems, cephalosporins, monobactams, and fluoroquinolones. ST463 carries different β-lactamase variants of class A (KPC), class B (IMP and AFM), and class D (OXA) (Table 1). The *bla*_KPC-2_ gene confers a high resistance to carbapenems and other β-lactams. *bla*_KPC-2_ was found in a plasmid named pPA1011 (ΔIS*6*Tn*3*-IS*Kpn*8-*bla*_KPC-2_-IS*Kpn*6-IS*26*) [80]. In addition, the transposon Tn6485f that carries both *bla*_IMP-45_ and *bla*_AFM-1_ is found in the plasmid pPA30_1. The plasmid pPA30_2 possesses two copies of the *bla*_KPC-2_ gene in the mobile element: IS*Kpn*27-*bla*_KPC-2_-IS*Kpn*6, and also in their chromosome carries the *aph* (3′)-IIb, *bla*_PAO_, *bla*_OXA-486_, *fosA*, *catB7*, and *crpP* [81,82]. More recently, the resistance to ceftazidime-avibactam (CZA) has been demonstrated in ST463 by the presence of *bla*_KPC-90_ [83] and *bla*_OXA-246_ [84]. Intermediate resistance to CST was reported in China [85]. ST463 emerges as a hypervirulent clone that carries the *exoU*^*+*^, *exoS*^*+*^, Phospholipase D-like effector (*pdlA*^*+*^) of the Type IV Secretion System and pyocyanin expression [81,86]. Thus, this clone can potentially spread and impact individuals with CF. This clone became a dominant endemic in eastern China and Korea.

## 5 Virulence of high-risk clones of *P. aeruginosa*

The emergence of high-risk clones of *P. aeruginosa* and their dissemination worldwide is a severe challenge for public health. Some clones increase their antimicrobial resistance by incorporating new mutations into their genome as a mechanism of microevolution and in response to aggressive environments [9]. Worldwide, some genetic variants were defined as high-risk clones (i.e., ST111, ST175, and ST235) [18,50]. Published studies using high-risk variants compared with reference strains show decreased virulence in high-risk clones (using cell lines or murine models). ST235, a multidrug resistance clone, mainly displays lower virulence than ST175 using the human type II alveolar epithelial cells A549 and the *Caenorhabditis elegans* model [87]. Another study shows in a peritonitis/sepsis murine model (C57BL/6 background) that ST235 is lethal in comparison with ST111 and ST175 [88]; in this same work, authors demonstrate that MDR strains were less virulent than non-MDR clinical isolates or reference strains (PAO1 and PA14). In addition, MDR strains induced less expression of TNFα and IL-10 than non-MDR clinical strains [89]; these data suggest that the adaptation process increases the antimicrobial resistance but decreases the host immune recognition.

The high-risk clone ST235 (genotype: *exoS*^*−*^*/exoU*^*+*^*)* was able to cause higher mortality in murine models and cell lines [45]. These results align with clinical observations that individuals infected with *P. aeruginosa* ST235 (*exoU*^+^) rapidly decrease lung function [90]. In addition, the presence of the *exoU* gene is considered a marker of high mortality risk [45]. In this regard, ExoU is considered a therapeutic target for developing new inhibitors [91]. Indeed, some reports mention that ST357, an isolate from Kyoto, Japan [92], the strain PA14 belonging to the ST253 [93], and ST463, an isolate from Zhejiang, China [81], also carry the *exoU* gene.

In conclusion, this mini-review work can be helpful in the medical and scientific community to understand how *P. aeruginosa* can adapt to diverse changing environments; this adaptation process is accompanied by constant incorporation and maintenance of mutations in genes associated with DNA repair. The mutator phenotype of *P. aeruginosa* is linked with the shift from acute to chronic infection during respiratory colonization. Indeed, new gene mutations coding for β-lactamases class A, B, or D can increase the spectrum of hydrolytic activity against new antimicrobials. Thus, the antimicrobial pressure can be another aggressive environment in which *P. aeruginosa* can promote the acquisition of new mutations. Several reports indicate that mutations in genes of DNA reparation downregulate the virulence factors' expression to favor chronic infections. Finally, we believe that knowledge of the mechanisms of antimicrobial resistance can help stablish new strategies to stop high-risk clones and make better decisions to eradicate such infections.

## CRediT authorship contribution statement

**Verónica Roxana Flores-Vega:** Writing – review & editing, Writing – original draft, Formal analysis, Conceptualization. **Santiago Partida-Sanchez:** Writing – review & editing, Conceptualization. **Miguel A. Ares:** Writing – review & editing, Conceptualization. **Vianney Ortiz-Navarrete:** Writing – review & editing, Conceptualization. **Roberto Rosales-Reyes:** Writing – review & editing, Writing – original draft, Supervision, Formal analysis, Conceptualization.

## Data availability statement

Data included in the article is referenced in the article.

## Funding

This work does not receive any founds.

## Declaration of competing interest

The authors declare that they have no known competing financial interests or personal relationships that could have appeared to influence the work reported in this paper.
